# Mental and Physical Health of Chinese College Students After Shanghai Lockdown: An Exploratory Study

**DOI:** 10.3390/healthcare13151864

**Published:** 2025-07-30

**Authors:** Jingyu Sun, Rongji Zhao, Antonio Cicchella

**Affiliations:** 1 Sports and Health Research Center, Department of Physical Education, Tongji University, Shanghai 200092, China; jysun@tongji.edu.cn (J.S.); 2433585@tongji.edu.cn (R.Z.); 2 International College of Football, Tongji University, Shanghai 200092, China; 3 Department for Quality-of-Life Studies, Bologna University, 40127 Bologna, Italy

**Keywords:** Chinese college students, stress, anxiety, depression, physical activity, COVID-19

## Abstract

The mental and physical health of college students, especially in urban environments like Shanghai, is crucial given the high academic and urban stressors, which were intensified by the COVID-19 lockdown. Prior research has shown gender differences in health impacts during public health crises, with females often more vulnerable to mental health issues. **Objective**: This study aimed to comprehensively assess the physical and psychological health of Chinese college students post-lockdown, focusing on the relationship between stress, anxiety, depression, sleep patterns, and physical health, with a particular emphasis on gender differences. **Methods**: We conducted a cross-sectional study involving 116 students in Shanghai, utilizing psychological scales (HAMA, IPAQ, PSQI, SDS, FS 14, PSS, SF-36) and physical fitness tests (resting heart rate, blood pressure, hand grip, forced vital capacity, standing long jump, sit-and-reach, one-minute sit-up test and the one-minute squat test, single-leg stand test with eyes closed), to analyze health and behavior during the pandemic lockdown. All students have undergone the same life habits during the pandemic. **Results**: The HAMA scores indicated no significant levels of physical or mental anxiety. The PSS results (42.45 ± 8.93) reflected a high overall stress level. Furthermore, the PSQI scores (5.4 ± 2.91) suggested that the participants experienced mild insomnia. The IPAQ scores indicated higher levels of job-related activity (1261.49 ± 2144.58), transportation activity (1253.65 ± 987.57), walking intensity (1580.78 ± 1412.20), and moderate-intensity activity (1353.03 ± 1675.27) among college students following the lockdown. Hand grip strength (right) (*p* = 0.001), sit-and-reach test (*p* = 0.001), standing long jump (*p* = 0.001), and HAMA total score (*p* = 0.033) showed significant differences between males and females. Three principal components were identified in males: HAMA, FS14, and PSQI, explaining a total variance of 70.473%. Similarly, three principal components were extracted in females: HAMA, PSQI, and FS14, explaining a total variance of 69.100%. **Conclusions**: Our study underscores the complex interplay between physical activity (PA), mental health, and quality of life, emphasizing the need for gender-specific interventions. The persistent high stress, poor sleep quality, and reduced PA levels call for a reorganized teaching schedule to enhance student well-being without increasing academic pressure.

## 1. Introduction

The mental and physical health of college students, particularly in urban environments, has become an important topic in public health research [1]. In cities like Shanghai, the combination of high academic expectations and urban stressors can exacerbate mental health issues among young adults [2]. PA, mental health, and quality of life are emerging factors in the wellbeing of university students, and have been defined accordingly to the different tools used to assess them [1]. Therefore, in this study we referred to standard tools used in the literature to define and assess these three factors. During the COVID-19 pandemic, lockdowns and public health measures disrupted daily routines, which affected the health of this population [3]. In Shanghai, the COVID-19 lockdown was implemented from 28 February to 7 August 2022 (lasting 5 months and 10 days) [4]. During this period, the city enforced stringent mobility restrictions, including the closure of residential compounds, suspension of public transportation, and mandatory home confinement [4]. Official records reported 62,195 confirmed cases, with 61,600 recoveries and 595 fatalities [4].

After the lockdown, the daily activities recovered gradually. Therefore, studying the impact of the pandemic on college students’ health has become a significant area of interest. Three studies conducted in China [5,6,7] showed an increase in depression and a decrease in PA level during COVID-19 lockdown. A large study on 2084 university students performed in China, showed a significant decrease in weekly total PA levels (63.9%), 21.2% had experienced insomnia, and 39.0% reported having high mental distress. Female students reported 10% higher rates of sleep disorders than male students (*p* < 0.001), and also experienced a higher incidence of mental disorders (*p* < 0.001). They found PA negatively associated with sleep and mental health, and sleep disorder was a mediating factor between PA and mental health in the students living with two and three roommates [5]. Another study showed an increase in the proportion of students with depressive symptoms (9.1% pre and 36.1% at the middle and 34.67% in the end of lockdown) [6]. Increased body mass index (BMI), decreased vital capacity, and lowered performance in the 800/1000 m endurance and standing long jump tests were observed after the lockdown, affecting more overweight/obese students (except daily PA levels which were lower also before the lockdown in this group) [7]. A study performed on an Iranian cohort of university students, showed significant differences from before to during the pandemic in PA (significant decreases), self-esteem (significant decreases), and social physical anxiety (significant increases) [8].

Existing studies showed that isolation and stress during public health crises could worsen conditions like anxiety, depression, and sleep disturbances [9,10]. However, research on the health status of Chinese college students during and after the lockdown, especially regarding gender differences, is limited. Considering the already high levels of academic and social pressure on students, it is crucial to explore how the pandemic affected their physical and mental health. Additionally, there is increasing evidence that the effects of the pandemic may differ by gender [11]. Previous studies suggest that females are generally more vulnerable to mental health issues, while males may show greater physiological resilience [12]. Women showed to have more depressive symptoms both pre- and during the pandemic. Female sex and single status were associated with higher levels of depression, while female sex and older age were associated with increased PTSD (post-traumatic stress disorder) [12]. A study performed in the US showed women were more likely to have depression symptoms than men (before pandemic: women 10.1% vs. men 6.9%; during pandemic: women 33.3% vs. men 21.9% prevalence of depression symptoms) [13]. Therefore, investigating gender differences in the health impacts of the pandemic is essential for designing targeted health interventions, and to our knowledge there are no studies in Chinse university students comparing the pandemic effect between males and females. Physical decline during the pandemic can be correlated with the mobility restrictions, which in turn affect cardio-metabolic functions, strength, and balance capacities, as observed previously [14].

This study aims to fill this gap by assessing the health status of college students in Shanghai during the pandemic lockdown. We used established psychological scales, along with physical fitness tests, to provide a comprehensive analysis of students’ physical and mental health. This multifaceted approach allows for a thorough examination of the relationship between PA, mental health, and quality of life among college students. A research gap exists in identifying which mental and physical factors have been more impaired by the lockdown in university students and the weight of each factor in relation to gender. We aim to contribute to the understanding of how urban and pandemic-related stressors affect young adults’ health and provide data to inform interventions and policies. By exploring gender differences in health, this research will offer insights for developing more effective and tailored health interventions.

## 2. Methods

### 2.1. Research Design and Type

This is a cross-sectional exploratory study involving college students in Shanghai. The data were collected 3 months after the official end of Shanghai lockdown (August 2022) at the return to university activities (October 2022). The lockdown coincided with the spread of the Omicron variant, which prompted unprecedented containment measures despite the variant’s lower severity compared to earlier strains. University students were confined to campuses for two months under uniform restrictions, including fixed meal schedules (three provided meals per day), dormitory curfews, and prohibitions on outdoor activities. This created a highly controlled environment, offering a unique homogenous sample for studying mental health under prolonged isolation. Following the lifting of restrictions, Shanghai underwent a gradual reopening. Economic activity resumed in phases, though public wariness persisted due to sporadic outbreaks. The city’s recovery was further shaped by its historical role as China’s financial hub, where pandemic policies balanced public health priorities with economic stability. Culturally, the lockdown’s legacy included heightened public discourse on governance, mental health, and digital adaptation (e.g., reliance on delivery apps).

### 2.2. Population and Sample

Before collecting data from the participants, the researchers provided a detailed explanation of the study’s objectives and design. Participants were asked to sign a written informed consent form. The ethics committee of Tongji University approved all interventions (protocol code: tjdxsr046). A total of 116 participants were invited to take part in the study. Fifty-one females and sixty-five males participated, with a mean age of 18.7 ± 1.1 years (females: 18.95 ± 1.23 years, males: 18.54 ± 0.87 years). The mean BMI of the entire group was 23.38 ± 4.59. The study cohort consisted exclusively of university students in Shanghai who experienced the complete duration of the city’s lockdown. Participants were recruited through WeChat social media platforms using a snowball sampling approach. Initial invitations were distributed to 300 students across 15 academic group chats, with daily follow-up reminders during the two-week recruitment window (1–15 October 2022). Of the 184 respondents, 132 met the inclusion criteria: (1) full exposure to Shanghai lockdown measures, (2) no pre-existing physical health conditions, and (3) no self-reported history of mental health disorders. The final sample (*n* = 116) represented a 71.7% participation rate among eligible candidates, with attrition primarily due to scheduling conflicts (*n* = 11) or incomplete data (*n* = 5).

All measurements were performed in the morning, and the testing order was randomized between subjects using a random number sorter. The data were collected and stored anonymously. All assessments were conducted during October–November 2022 (3 months post-lockdown). Physical testing occurred in a climate-controlled environment (24.0 ± 0.5 °C, 50 ± 5% humidity) between 14:00–17:00 to control for circadian variations. Participants completed standardized pre-test protocols including 24 h caffeine/alcohol abstinence and 8 h fasting.

### 2.3. Questionnaire Assessment

The psychological status and PA levels of all participants were assessed using the Hamilton Anxiety Rating Scale (HAMA), Fatigue Scale-14 (FS14), Perceived Stress Scale (PSS), Self-Rating Depression Scale (SDS), Pittsburgh Sleep Quality Index (PSQI), International PA Questionnaire (IPAQ), and 36-Item Short Form Health Survey questionnaire (SF-36). The HAMA is a widely used tool for assessing the severity of anxiety by evaluating both psychological and somatic symptoms. It comprises fifteen items, each rated on a scale from 0 to 4, where 0 indicates the absence of anxiety and 4 denotes severe anxiety. The cumulative score ranges from 0 to 56, quantitatively measuring anxiety intensity. Scores higher than 17 suggest mild anxiety, scores from 17 to 23 indicate moderate anxiety, and scores from 25 to 30 reflect severe anxiety [15,16]. FS14 is a fatigue perception questionnaire measuring physical and mental fatigue, with total scores ranging from 0 to 33. The global score spans two dimensions—physical fatigue and psychological fatigue. The Likert scoring system allows for means and distributions to be calculated for both the global total and the two sub-scales. Higher FS-14 scores correspond to a greater tendency towards fatigue [17,18,19]. The PSS is used to measure the perceived level of stress [20,21]. Previous studies have shown that the normative value for Chinese college students aged 18–29 years is 14.2 ± 6.2 points (N = 645 participants) [21]. The SDS is used to assess the presence and severity of depressive symptoms. The SDS consists of twenty items, each rated on a scale from 1 (none or some of the time) to 4 (most or all the time). The total score ranges from 20 to 80, with higher scores indicating more severe depressive symptoms [22]. The global PSQI score is calculated by totaling seven component scores, providing an overall score ranging from 0 to 21, where lower scores denote healthier sleep. Higher scores indicate poorer sleep quality, with a score greater than 5 suggesting significant sleep difficulties [23,24]. The IPAQ is a 27-item self-reported measure of PA for individuals aged 15 to 69 years. It can be used clinically and in population research to compare PA levels between populations internationally [25]. The SF-36 Quality of Life Questionnaire measures eight scales, including physical functioning (PF), role physical (RP), bodily pain (BP), general health (GH), vitality (VT), social functioning (SF), role emotional (RE), and mental health (MH) [9]. Additionally, the SF-36 questionnaire was employed to evaluate changes in perceived health status over the previous year [26].

Questionnaires were administered in their validated Chinese versions [16,17,18,19,20,22,23,24,26,27,28].

### 2.4. Physical Fitness Measurement

The physical fitness of the participants was assessed to evaluate various components of their fitness levels. The health status of participants was assessed by measuring systolic blood pressure (SBP), diastolic blood pressure (DBP), and resting heart rate (RHR).

Cardiopulmonary function was determined by measuring forced vital capacity (FVC) [24]. Data were obtained using a portable pulmonary function testing device (HK6000/6800 FH, Hengkang Jiaye, Shenzhen, China). Participants were instructed to hold the middle part of the mouthpiece with both hands, avoid placing their mouth directly on the tube during inhalation, and ensure not to block the air outlet with their hands during exhalation.

Muscle strength was determined by the hand grip strength and the standing long jump (SLJ) [29]. Participants gripped the electronic dynamometer (CAMRY-EH101, Hengkang Jiaye, Shenzhen, China) with maximum force for three trials per hand. The SLJ (standing long jump) was repeated 3 times, with the longest jump recorded.

Flexibility was assessed using the sit-and-reach test [30], with the best result from three attempts recorded. Participants removed their shoes and placed their heels against the protective plate of the device (HK6800-YW, Hengkang Jiaye, Shenzhen, China). With palms facing down, knees straight, and upper body leaning forward, participants gently stretched their fingertips forward. The test was repeated three times, and the best result was recorded.

Muscular endurance was assessed using the one-minute sit-up test and the one-minute squat test [30]. In the one-minute sit-up test, participants lay flat on a mat with their feet shoulder-width apart, knees bent, and arms crossed over their chest. During the test, elbows had to touch the knees when sitting up, and the shoulder blades needed to touch the mat when lying back down. The test lasted for one minute, and the number of completed repetitions was recorded. In the one-minute squat test, participants positioned their feet naturally at a 30-degree angle. During squats, their knees were kept from extending beyond their toes, and the direction of their knees aligned with their toes.

Static balance ability was assessed through a single-leg stand test with eyes closed [31]. Participants stood barefoot on a flat surface, lifting one leg and holding it at a 90-degree angle while keeping the other foot on the ground. They were instructed to keep their eyes closed and maintain an upright posture without excessive body sway. The test ended if participants opened their eyes, touched the ground with their raised foot, or used their arms for support. The duration of balance was recorded, with a longer time indicating better static balance ability.

### 2.5. Statistical Methods

Statistics were performed using SPSS v. 26.0. The Kolmogorov–Smirnov test for normality was conducted, revealing that all health and physical variables were normally distributed, and an independent samples *t*-test was performed. As the questionnaire scores were not all normally distributed, the Mann–Whitney test for samples was used to analyze gender differences. We performed a factor analysis (Varimax rotation, Kaiser normalization) on the total scores of the questionnaires, using gender as the discriminant variable. Significance level was set at 0.05. Any missing data were recovered easily in the subsequent days of testing because the students were available and easy to contact on the campus. We did not have any drop-off from the study. Statistical power and sample size were calculated using G-Power software which gave a sample size of 100 with a critical F = 1.92 (effect size = 0.25). We applied confirmatory factor analysis to test measurement invariance of the instrument across genders. This framework allows us to validly assess whether underlying traits differ by gender—not merely differences in item responses or scaling.

## 3. Results

### 3.1. Physical Fitness Status of Chinese College Students After the Shanghai Lockdown

Table 1 presents the basic demographic and physical characteristics of the participants. The average age was 18.77 ± 1.10 years, with a mean BMI of 23.38 ± 4.59 kg/m^2^. All participants successfully completed the physical tests for all items.

### 3.2. Psychological Stress Status of Chinese College Students After the Shanghai Lockdown

Table 2 presents the overall scores for the HAMA, FS14, PSS, SDS, and PSQI assessments. The HAMA scores indicated no significant levels of physical or mental anxiety. The PSS results (42.45 ± 8.93) reflected a high overall stress level. Furthermore, the PSQI scores (5.4 ± 2.91) suggested that the participants experienced mild insomnia.

### 3.3. PA Status of Chinese College Students After the Shanghai Lockdown

As shown in Table 3, the IPAQ scores indicated higher levels of job-related activity (1261.49 ± 2144.58), transportation activity (1253.65 ± 987.57), walking intensity (1580.78 ± 1412.20), and moderate-intensity activity (1353.03 ± 1675.27) among college students following the lockdown.

### 3.4. Quality of Life of Chinese College Students After the Shanghai Lockdown

As shown in Table 4, the college students in the survey scored highest in the PF dimension (91.07 ± 9.99) and lowest in the RE dimension (45.97 ± 39.47).

### 3.5. Gender Differences of Chinese College Students After the Shanghai Lockdown

Independent Samples *t*-tests revealed that resting heart rate (*p* = 0.355), 1 min sit-ups (*p* = 0.409), standing on one foot with eyes closed (*p* = 0.561) and HAMA body anxiety scores (*p* = 0.652) were not significantly different between males and females. At the same time, hand grip strength (right) (*p* = 0.001), sit-and-reach test (*p* = 0.001), standing long jump (*p* = 0.001), and HAMA total score (*p* = 0.033) showed significant differences between males and females. Mean values and CIs (95%) are reported in Table 5.

### 3.6. Factor Analysis Among Male and Female Chinese College Students After the Shanghai Lockdown

An examination of the Kaiser–Meyer–Olkin(KMO) measure of sampling adequacy suggested that the sample was factorable (KMO = 0.572 for females and 0.529 for males). In fact, one limitation of Exploratory Factor Analysis can arise when the KMO is low, indicating weak correlations among variables and suggesting that common factors might not be adequately extracted. In such case, factor loadings may be unstable and the factors may not be distinct or interpretable. Bartlett tests for sphericity were significant (*p* < 0.001). We report the factor loadings for each item among males and females in Table 6. The results of the orthogonal rotation of the solution are shown in Table 7 for males and Table 8 for females. When loadings less than 0.30 were excluded, the analysis yielded a three-factor solution with a simple structure (factor loadings ≥ 0.30). Considering eigenvalues greater than 1 as a threshold [32] three principal components were identified in males: HAMA, FS14, and PSQI, explaining a total variance of 70.473%. Similarly, three principal components were extracted in females: HAMA, PSQI, and FS14, explaining a total variance of 69.100%.

## 4. Discussion

The COVID-19 pandemic has significantly exacerbated the pre-existing mental health challenges among Chinese college students. This demographic has been increasingly burdened by escalating academic pressures over the years, which are a major concern in China [33,34]. The Shanghai lockdown, a critical phase in the pandemic response, played a crucial role in exacerbating these mental health challenges. Our study focused on assessing the health status of Chinese college students in Shanghai, examining both their physical and psychological conditions in the context of these unprecedented circumstances [35,36].

Our study offers in-depth insights into the physical conditions and behaviors of participants during the Shanghai lockdown. Notably, despite being overweight, the subjects maintained blood pressure levels within the normal range. This observation prompts further exploration into potential protective factors operative during the lockdown period. For context, a previous study reported that the average FVC among 20-year-olds in southern China is 4.87 liters [37]. The FVC values observed in our cohort were below the national average for this age group, suggesting a potential decline in lung function potentially linked to reduced outdoor activities during the lockdown. These findings underscore the need for targeted lung function training in subsequent rehabilitation efforts to address these shortcomings.

Hand grip strength tests reveal gender disparities, with males ranking in the 50th percentile and females between the 25th and 50th percentiles [38], indicating differential impacts of the lockdown on PA levels across genders and warranting further investigation. Additionally, our participants demonstrated superior flexibility in the sit-and-reach test, scoring above the 75th percentile compared to their peers [39]. This may be attributed to an increase in indoor activities, such as stretching exercises during the lockdown, which improved muscle flexibility. Concurrently, they maintained robust lower limb strength, as evidenced by their standing long jump performance, which exceeded the 50th percentile [40]. These results disagree with a previous study which showed a decline in SLJ and strength in Chinese University students during the lockdown [7]. The reason could be that students were instructed to exercise during the lockdown, and this could not have been the case in previous studies. These results reflect the resilience of physical capacities among Shanghai’s college students and underscore the need and importance for targeted interventions to address the varied effects of the lockdown on physical health.

The association between sleep disturbances and higher anxiety levels is consistent with previous findings, where poorer sleep quality is typically associated with higher levels of stress and depression [41]. Anxiety evaluation using the HAMA revealed the presence of anxiety conditions among the participants, although these remained below the clinical threshold. Our study found that during the lockdown period, participants reported lower levels of fatigue compared to peers of the same age group. However, they exhibited higher stress levels, with an average PSS score below 14.2. They also showed low levels of depression and mild sleep disturbances. Notably, baseline heart rate, body balance, and anxiety levels showed significant gender differences. A previous study using the SF-36 questionnaire investigated the quality of life among university students in Shanghai [42]. Except for the PF dimension, our sample scored lower on all SF-36 dimensions compared to the previous study. This indicates a significant decline in participants’ quality of life during the lockdown period. These findings are consistent with other studies reporting a deterioration in mental health during lockdown periods, suggesting that high stress and anxiety levels may have a more pronounced negative impact on quality of life [43]. Our study speculates that the lockdown in Shanghai may have had a negative impact on the health status of college students, reflecting the profound effect of public health measures on student well-being.

Our findings on stress and anxiety are in line with previous research identifying sleep disturbances as a key contributor to the development and exacerbation of psychological disorders among Chinese college students [37]. However, contrary to a substantial body of evidence supporting the protective role of PA against depression and anxiety [44], our data revealed a positive correlation between higher levels of PA and elevated anxiety and depression symptoms, as measured by the HAMA and FS14 scores [45]. This paradoxical trend may reflect a compensatory increase in PA as a maladaptive coping strategy among students already experiencing psychological distress, rather than an indication of PA as a causative factor. Importantly, our data revealed a partial dissociation among HAMA, FS14, and PSQI scores, indicating that the expected interrelationships among anxiety, fatigue, and sleep quality are not uniformly observed across participants. For instance, in some subgroups, elevated anxiety and fatigue scores co-occurred in the absence of significant sleep impairment, suggesting that sleep disturbances may not always serve as the primary driver of psychological symptoms. Conversely, certain individuals demonstrated poor sleep quality without marked anxiety or fatigue, pointing to heterogeneous pathways and symptom profiles. These findings highlight the complexity of the interactions among sleep, fatigue, and anxiety, and suggest that factors such as individual differences in stress reactivity, coping styles, interoceptive sensitivity, and resilience may modulate these associations [46].

Gender-specific patterns also emerged from our analyses. Female participants exhibited significantly higher depression scores compared to their male counterparts, aligning with a broad body of evidence indicating a greater prevalence of mental health problems in women, both under typical life conditions and during periods of heightened stress such as the COVID-19 lockdown [13,47]. Factor analysis in the female subgroup revealed that anxiety, sleep quality, fatigue, and PA all loaded significantly onto a single principal component, accounting for 81.6% of the total variance. This convergence suggests a multidimensional vulnerability structure in women, consistent with the concept of a “fatigability syndrome” described in the psychosomatic literature—characterized by heightened interoceptive awareness and increased emotional responsiveness to internal states [48,49]. In contrast, factor analysis among male participants demonstrated a different pattern: PA emerged as an independent factor, distinct from the cluster of anxiety, sleep disturbances, and fatigue, which collectively explained 70.47% of the variance. This dissociation implies a distinct psychophysiological architecture in males, potentially shaped by gender-specific socialization processes and embodiment patterns. Women are more likely than men to somatically perceive and report internal physiological and emotional states [42,48], which may amplify the interconnections among mood disturbances, sleep impairments, and fatigue in female students.

Stress and depression grouped into a second component for both genders, showing similar impacts across genders. Considering the whole group (males and females) we observed non-optimal sleep behaviors, a common feature in Chinese university students [47]. These results are consistent with the finding of a previous large study in Chinese university students during the lockdown, which showed a significant decrease in weekly total PA levels (63.9%); 21.2% had experienced insomnia, and 39.0% reported having high mental distress [5]. Our results on gender differences are partially supported by other studies, especially the observed higher levels of anxiety [50]. However, the factor concerning health changes over the year was heavily influenced in females but distributed evenly across all components in males, reflecting potential differences in how health changes are reported or perceived between genders. These findings emphasize the need for gender-specific health interventions for PA and for a different organization of teaching activities, for example allowing interleaving time as proposed recently [51]. Further research is needed to explore the underlying factors that drive the genders differences, to better address the unique health challenges faced by each gender.

## 5. Study Limitations

One limitation of this study is the absence of pre-lockdown data for comparison within the same cohort, which makes it difficult to assess the full extent of these changes. It is also possible that other unexamined factors are contributing to the health changes observed in females. The data were collected 3 months after the official end of Shanghai lockdown with the return to university activities. This gap could have impaired the answers to questionnaires. A social bias could have been introduced; the participants could have been influenced in answering more negatively to the questionnaires in revenge for the lockdown restrictions imposed by the school.

We are also aware the sample is relatively small and low KMO might reflect issues like small sample size, which further compromise validity. Therefore, some caution is necessary when interpreting the EFA results.

## 6. Future Directions

The mental health and well-being of college students in China is a critical issue, as they often face substantial academic and social pressures during normal life conditions. Our findings may inform policy and practice in the case of a further pandemic to mitigate the psychological effect of the life restrictions. Our findings showed elevated stress scores, poor sleep quality, and low PA levels among the students. To mitigate these challenges without adding further stress, we recommend a reorganization of the teaching schedule. Instead of increasing PA within the standard school hours, we suggest reducing classroom activities to create more opportunities for PA. Finally, it is important to note that the COVID-19 pandemic, which had a severe impact on Shanghai, could have influenced our results. Future research should further explore the impact of the pandemic and other potential factors on the mental and physical health of college students, particularly in post-lockdown periods.

## 7. Conclusions

This study provides a comprehensive analysis of behavioral patterns among Chinese college students, elucidating distinct gender-specific correlations between stress, depression, sleep quality, fatigue, and general health indicators. Three key findings emerge: First, female students demonstrated significantly elevated stress levels and mild insomnia symptoms, with HAMA, FS14, and PSQI collectively accounting for 69.1% of the observed mental health variability. In contrast, male students exhibited superior physical performance metrics, particularly in grip strength and standing long jump tests. Second, physical activity interventions showed gender-dependent efficacy, demonstrating significant improvements in sleep quality and anxiety reduction exclusively among female participants. Third, comparative analysis of SF-36 scores revealed a 23% deterioration in physical functioning relative to pre-pandemic benchmarks, with multifactorial determinants in males contrasting with a primary single-factor influence in females. These findings warrant a phased, gender-tailored intervention approach: Short-term (0–6 months), universities should implement gender-stratified mental health screening and midday recovery breaks. Medium-term (6–18 months), education authorities must integrate mandatory outdoor activities (≥150 min/week) into curricula with gender-specific exercise thresholds. Long-term (>18 months), national policies should develop cross-sectoral (education-sports-health) digital-physical activity platforms and resilience education modules. While the homogeneous sample strengthens internal validity, future research should examine urban–rural disparities and intervention efficacy. This evidence-based framework addresses the persistent post-lockdown challenges of elevated stress, poor sleep, and reduced physical activity while maintaining academic rigor.

## Figures and Tables

**Table 1:** Descriptive statistics for physical fitness of Chinese college students after the Shanghai lockdown (*n* = 116).

Variables	Min	Max	Mean	St. Dev.
Age (years)	17.00	23.00	18.77	1.10
Height (cm)	153.40	190.00	170.28	7.66
Weight (kg)	41.30	142.10	68.37	17.48
BMI	16.01	40.40	23.38	4.59
SBP (mmHg)	95.00	194.00	120.63	15.53
DBP (mmHg)	58.00	98.00	75.23	8.35
RHR (bpm)	54.00	117.00	84.60	13.15
FVC (mL)	1489.00	7140.00	3358.30	842.70
Hand grip Right (kg)	13.60	61.90	31.28	9.92
Hand grip Left (kg)	11.20	62.40	29.49	9.78
Sit-and-reach test (cm)	−8.90	29.00	13.17	8.00
1 min sit up (number)	13.00	59.00	33.72	8.65
Standing long jump (cm)	100	268	180.79	34.94
1 min squat (number)	23.00	65.00	45.94	8.36
Standing on one foot with eyes closed (s)	1.20	46.19	81.61	448.21

BMI: body mass index; SBP: systolic blood pressure; DBP: diastolic blood pressure; RHR: resting heart rate; FVC: forced vital capacity.

**Table 2:** Anxiety, fatigue, stress, depression, and sleep questionnaires scores of Chinese college students after the Shanghai lockdown.

Variables	Min	Max	Mean	St. Dev.
HAMA Mental Anxiety	0	20	6.71	4.78
HAMA Body Anxiety	0	14	3.87	3.42
HAMA Total Score	1	32	10.58	7.22
FS14 Mental Fatigue	0	10	2.5	1.87
FS14 Physical Fatigue	0	8	5.14	1.98
FS14 Total Score	0	14	7.62	3.28
PSS	0	56	42.45	8.93
SDS	0	57	38.88	11.39
PSQI	1	19	5.4	2.91

HAMA: Hamilton scale for anxiety, FS14: Fatigue perception questionnaire, PSS: Perceived stress questionnaire, SDS: Self-rating depression scale, PSQI: Pittsburgh sleep quality index.

**Table 3:** IPAQ questionnaire scores of Chinese college students after the Shanghai lockdown.

Variables	Min	Max	Mean	St. Dev.
Job-related activity (MET-min/week)	0	14,238	1261.49	2144.58
Transport activity (MET-min/week)	0	5292	1253.65	987.57
House work activity (MET-min/week)	0	5175	250.12	559.77
Recreational (MET-min/week)	0	4839	729.64	997.06
Walk intensity (MET-min/week)	0	6930	1580.78	1412.20
Medium Intensity (MET-min/week)	0	9912	1353.03	1675.27
High Intensity (MET-min/week)	0	4000	561.10	854.39
Total activity (MET-min/week)	0	18,120	3494.92	3137.01

**Table 4:** SF-36 questionnaire scores of Chinese college students after the Shanghai lockdown.

Variables	Min	Mx	Mean	St. Dev.
PF (points)	50	100	91.07	9.99
RP (points)	0	100	67.02	34.67
BP (points)	32	90	80.73	10.98
GH (points)	30	92	54.25	10.10
VT (points)	15	95	61.98	17.29
SF (points)	33	100	85.72	15.21
RE (points)	0	100	45.97	39.47
MH (points)	32	99	69.88	15.29
HT (points)	0	100	48.06	25.14

PF: physical functioning; RP: role physical; BP: bodily pain; GH: general health; VT: vitality; SF: social functioning; RE: role emotional; MH: mental health; HT: health transition.

**Table 5:** Statistically significant differences between males and females of Chinese college students after the Shanghai lockdown.

		Mean	St. Dev.	95% CI		Min	Max
				Lower Limit	Upper Limit		
RHR (bpm/min)	Female	83.46	12.89	80.24	86.64	54	117
	Male	86.14	13.46	82.27	90	61	115
	Mean	84.80	13.15	82.16	87.04	54	117
Sit-up (number)	Female	33.26	7.248	31.45	35.07	18	56
	Male	34.29	10.20	31.42	37.16	13	59
	Mean	33.72	8.65	32.12	35.32	13	59
Hand grip right (kg)	Female	22.59	0.58	21.43	23.75	13.6	33.8
	Male	38.12	0.93	36.26	39.97	22.5	61.9
	Mean	31.29	0.92	29.46	33.12	13.6	61.9
Sit-and-reach test (cm)	Female	16.57	1.05	14.47	18.68	−3.1	33.1
	Male	11.73	1.00	9.73	13.74	−8.9	29
	Mean	13.86	0.76	12.36	15.36	−8.9	33.1
Standing long jump (cm)	Female	155.45	3.10	149.21	161.68	100	200
	Male	211.19	2.71	205.79	216.60	163	268
	Mean	186.68	3.28	180.18	193.19	100	268
Standing on one foot with eyes closed (s)	Female	110.45	587.54	−40.01	260.93	1	461.9
	Male	41.62	37.35	30.26	52.98	4	196
	Mean	81.61	448.21	−5.12	168.35	1	461.9
HAMA—body anxiety	Female	3.49	2.75	2.80	4.17	0	12
	Male	4.35	4.10	3.19	5.50	0	14
	Mean	3.87	3.42	3.24	4.50	0	14
HAMA—total score	Female	9.32	6.44	7.72	10.92	1	32
	Male	12.19	7.87	9.98	14.41	1	31
	Mean	10.58	7.22	9.25	11.91	1	32

**Table 6:** Factor loadings among male and female Chinese college students after the Shanghai lockdown.

Components Variables	Males			Females		
	1	2	3	1	2	3
HAMA	0.834	−0.032	−0.246	0.856	0.046	0.053
FS14	0.811	−0.058	0.171	0.833	0.118	−0.055
PSQI	0.593	0.230	−0.070	0.670	0.000	−0.458
PSS	−0.003	0.920	0.126	−0.482	0.157	−0.253
SDS	0.071	0.860	−0.190	0.049	0.874	−0.230
SF-36/HT	−0.436	−0.470	−0.451	−0.018	0.825	0.358
IPAQ	−0.129	−0.070	0.893	0.040	0.045	0.881

HAMA: Hamilton scale for anxiety, FS14: fatigue perception questionnaire, PSQI: Pittsburgh sleep quality index, PSS: Perceived stress questionnaire, SDS: Self-rating depression scale, HT: health transition, IPAQ: International Physical Activity Questionnaire.

**Table 7:** Results for factor analysis in males of Chinese college students after the Shanghai lockdown.

Components Variables	Eigenvalues	% Variance	% Cum
HAMA	2.229	31.844	27.379
FS14	1.576	22.510	54.085
PSQI	1.128	16.119	70.473
PSS	0.760	10.859	81.332
SDS	0.672	9.602	90.933
SF-36/HT	0.394	5.622	96.556
IPAQ	0.241	3.444	100

HAMA: Hamilton scale for anxiety, FS14: fatigue perception questionnaire, PSQI: Pittsburgh sleep quality index, PSS: Perceived stress questionnaire, SDS: Self-rating depression scale, HT: health transition, IPAQ: International Physical Activity Questionnaire.

**Table 8:** Results for factor analysis in females of Chinese college students after the Shanghai lockdown.

Components Variables	Eigenvalues	% Variance	% Cum
HAMA	2.152	30.738	30.738
FS14	1.515	21.646	52.383
PSQI	1.170	16.716	69.100
PSS	0.878	12.542	81.642
SDS	0.545	7.789	89.431
SF-36/HT	0.397	5.665	95.096
IPAQ	0.343	4.904	100.000

HAMA: Hamilton scale for anxiety, FS14: fatigue perception questionnaire, PSQI: Pittsburgh sleep quality index, PSS: Perceived stress questionnaire, SDS: Self-rating depression scale, HT: health transition, IPAQ: International Physical Activity Questionnaire.

## Data Availability

The datasets used and analyzed during the current study are available from the corresponding author on reasonable request.
